# Dosimetry for targeted radionuclide therapy in routine clinical practice: experts advice vs. clinical evidence

**DOI:** 10.1007/s00259-023-06568-8

**Published:** 2023-12-19

**Authors:** Arnaud Dieudonné, Clément Bailly, Florent Cachin, Agathe Edet-Sanson, Françoise Kraeber-Bodéré, Sébastien Hapdey, Charles Merlin, Philippe Robin, Pierre-Yves Salaun, Paul Schwartz, David Tonnelet, Pierre Vera, Frédéric Courbon, Thomas Carlier

**Affiliations:** 1 Department of Nuclear Medicine, Henri Becquerel Cancer Center, Rouen, France; 2 Service de Médecine Nucléaire, Centre Henri Becquerel, 76000 Rouen, France; 3 Department of Nuclear Medicine, University Hospital, Nantes, France; 4 Department of Nuclear Medicine, Jean Perrin Cancer Center, Clermont-Ferrand, France; 5 Department of Nuclear Medicine, University Hospital, Brest, France; 6 Department of Nuclear Medicine, University Hospital, Bordeaux, France; 7 Department of Medical Imaging, Institut Universitaire du Cancer Toulouse - Oncopole, Toulouse, France

The European Federation of Organisations for Medical Physics (EFOMP) has recently published a statement on the role of dosimetry in radionuclide therapy (RNT) [1]. This statement relies on article 56 of the European Council directive 2013/59/Euratom to advocate the need for personalized dosimetry to plan and monitor RNT. As a reminder, this Euratom article states that “For all medical exposure of patients for radiotherapeutic purposes, exposures of target volumes shall be individually planned and their delivery appropriately verified taking into account that doses to non-target volumes and tissues shall be as low as reasonably achievable (ALARA) and consistent with the intended radiotherapeutic purpose of the exposure.” It is important to bear in mind that nuclear medicine physicians do not prescribe Gray to a target but administer a ponderable dose in Bequerels of a human medical product delivered by a radiopharmacist. Article 1 of Directive 2001/83/EC defines a “medicinal product” as “Any substance or combination of substances presented as having properties for treating or preventing disease in human beings; Any substance or combination of substances which may be used in, or administered to, human beings, either with a view to restoring, correcting or modifying physiological functions by exerting a pharmacological, immunological or metabolic action, or to making a medical diagnosis”. Contrary to what was claimed years ago by some of the authors of the EFOMP statement [2], there is no conflict between the registration of radiopharmaceuticals and the Euratom Directive. There is no contraindication to dosimetry-based posology in the European regulation relative to medicinal products as long as it is supported by clinical data.

We therefore agree with the position of the European Association of Nuclear Medicine, proposing different levels of compliance with the directive depending on the treatment modality [3]. There is indeed a difference in the level of evidence for the 2 types of RNT, i.e., selective internal radiation therapy (SIRT) and radiopharmaceutical therapy (RPT), also referred to as targeted radionuclide therapy (TRT). In contrast to SIRT, the clinical benefit of dosimetry in RPT is not yet demonstrated despite some dose–effect correlations highlighted. Without a proven clinical benefit, a modification of the regimen of an approved drug outside of a clinical trial, either based on dosimetry or not, is defined as an off-label use. In the absence of safety and efficacy data, the patients must be informed and give their consent, while the prescription should clearly be mentioned as off-label. While physicians are used to prescribing drugs off-label, the practice is highly supervised, at least in France, and the personal responsibility of physicians is involved if adverse events occur. Off-label prescription should be exceptional and used only if there is no other option.

SIRT with microspheres is currently the only RNT modality where dosimetry has a proven benefit in terms of clinical outcome, i.e., tumor control probability (TCP) [4–6], non-tumor complication probability (NTCP) [7], and months of progression-free survival (PFS) or overall survival (OS). These results were obtained thanks to two clinical trials, one retrospective analysis [5] and one prospective with dosimetry as the primary objective [8]. In the 2000s, the body surface area (BSA) method and single-compartment dosimetry were recommended for resin and glass microspheres [9]. The turning point came in the early 2010s, when retrospective studies showing promising results were published [10, 11] and clinical investigation was promoted in a context where the respective resin and glass microsphere manufacturers competed for market share. International recommendations now propose treatment planning strategies based on dosimetry objectives depending on the treatment strategy and the clinical endpoint [12–14]. Scientific and clinical pieces of evidence, as well as patients interests, have prevailed in this context, demonstrating that the role of dosimetry cannot be reduced to a pros and cons debate.

Do we have clinical data to guide treatment planning for RPT? It appears that although dose–effect relationships are well documented, a clear clinically proven benefit in terms of TCP, NTCP, or months of survival is still pending [15].

For example, the correlation between bone marrow absorbed dose and platelets has been shown in radioiodine treatments [16], [^177^Lu]Lu-DOTATATE in neuroendocrine tumors (NET) [17, 18], and [^177^Lu]Lu-PSMA in metastatic castration-resistant prostate cancer (mCRPC) [19]. However, it has not yet been shown to improve treatment outcomes. Until proven otherwise, blood cell counts are an economically and clinically more efficient technique than dosimetry to monitor RPT bone marrow toxicity. Not to mention that grade 3 bone marrow toxicity remains rare [17, 20, 21] except in patients already heavily treated with chemotherapy in whom the capacities of hematopoietic regeneration seemed more limited and who presented more difficulties recovering from myelodysplastic disease [22, 23]. Furthermore, regardless of the bone marrow absorbed doses, the non-responding or even progressive disease itself seems to be one, if not the most important, factor for bone marrow impairment [24].

The kidney is another organ that has been investigated for dose–effect relationships with ^177^Lu-DOTATATE and [^177^Lu]Lu-PSMA. The results of the ILUMINET trial [25] suggested that the treatment of NET with [^177^Lu]Lu-DOTATATE could be monitored with renal dosimetry, but achieved similar PFS and OS than the NETTER-1 trial [21, 26] which was conducted with a standard regimen. Another study has implemented a treatment regimen based on renal dosimetry [27] by increasing the injected activity up to the presumed maximum tolerated renal absorbed dose, which did not improve the PFS compared to the aforementioned studies. In patients treated with [^177^Lu]Lu-PSMA, a recent retrospective study reported 3 cases of radiation-induced nephropathy following extensive treatment with [^177^Lu]Lu-PSMA [28]. While authors argued that individual dosimetry might have helped prevent these events, it is important to note that those patients received from eight to ten cycles with substantially higher cumulative activity than patients in the VISION trial (maximum 6 treatment cycles) [20] or patients in real life data (median of three to four cycles) and that the incidence of this toxicity remains extremely low (< 1%) and has not been found in the literature in similar populations [29]. In addition, this exceptionally high number of treatment cycles can only be seen in the absence of alternative treatment options, in a situation where the risk of chronic kidney disease is outweighed by the risk of tumor progression. These results illustrate well that although dose–effect relationships have been demonstrated, dosimetry is not the only parameter influencing the treatment outcome.

Regarding lesion dosimetry, it is associated with blood prostate–specific antigen (PSA) [30] for [^177^Lu]Lu-PSMA and with radiological response [31] for [^177^Lu]Lu-DOTATATE, but not with PFS or OS. As suggested by Alipour et al. [31], there are other factors than tumor response and absorbed dose that influence PFS and OS in NET. This may explain the dichotomy between RPT and SIRT. Indeed, SIRT is a loco-regional treatment for locally advanced or early-stage disease; therefore, local disease control, which correlates well with dosimetry, is strongly correlated with survival.

Another explanation could be that standard RPT regimens have been validated in clinical trials with a positive risk–benefit balance. The search for a clinical benefit from dosimetry may upset this balance if the tolerance limit of an organ is sought, with no guarantee of improved efficacy.

Nuclear medicine centers are facing a systemic and global shortage of healthcare professionals [32], in a context of increasing RPT [33] and stable healthcare costs per gross domestic product, except in the COVID era [34]. RPT healthcare workers (nuclear medicine physicians, radiopharmacists, medical physicists, technologists, etc.) should focus on clinically relevant tasks that have a proven benefit to the patient, i.e., dosimetry for SIRT, implementation of new RPT techniques, improvement of treatment follow-up based on imaging biomarkers, etc.

We should harness our multidisciplinary energy and apply for more funding to conduct more clinical trials with ancillary research to optimize this therapeutic option and improve patient outcomes. Cancer is becoming a chronic disease, so we need to manage time by limiting toxicities by trying to de-escalate and optimize drug treatments and ionizing irradiation through multidisciplinary research in physics, pharmacokinetics, dosimetry, and radiobiology.

Clinical dosimetry is one tool among others (quantification, radiomics, dynamic imaging, etc.) in the field of theragnostics. There is a potential for dosimetry to contribute to the development of new therapeutic strategies, new radiopharmaceuticals, new indications, etc., but this needs to be supported by clinical evidence [35] and should be done in a cost-effective manner. As recently stated by the Medical Physics Department of the Memorial Sloan Kettering Cancer Center, “If dosimetry is to become more than an academic exercise, we need to show that it makes a significant difference to clinical outcomes with RPT” [36]. A similar debate has been raging among oncologists and hematologists since the advent of chemotherapies and now targeted therapies, on the need for adaptive or individualized rather than fixed dosing. Only irrefutable clinical evidence of the crucial role of therapeutic drug monitoring and pharmacokinetics or pharmacodynamics analysis in the optimization of antineoplastic regimens could balance the economic and logistic complexity of this technique implementation in clinical routine [37–39]. This debate has marked the entire history of medicine. Although important in itself, expert opinion provides the weakest level of evidence; randomized prospective phase III trials with independent safety and efficacy data monitoring boards are the gold standard for assessing the clinical benefit of a new drug or new indication. It is then up to the regulatory authorities to carefully review the data and audit the trial sponsors and investigational sites for approval.
